# Trans-cellular transport of short chain fatty acids in the large intestine

**DOI:** 10.1186/1471-2164-15-S2-P4

**Published:** 2014-04-02

**Authors:** Taoufik Nedjadi

**Affiliations:** 1 King Fahd Medical Research Centre, King Abdulaziz University, Jeddah 21589, Kingdom of Saudi Arabia

## 

Short chain fatty acids (SCFA) are the main end-products of anaerobic degradation of dietary fiber in the colon [1]. SCFA contribute immensely to the body’s energy requirement and regulate numerous cellular processes during both health and disease [2]. SCFA especially butyrate possess anti-cancer activity through induction of apoptosis and promotion of differentiation by modulating the transcription of a number of underlying genes [3]. The exact transport mechanism of SCFA remains ambiguously defined, Hence we aim here to characterize the mechanism of butyrate transport across both luminal and basolateral membranes of the colonic epithelial cells.

## Materials and methods

Luminal membrane vesicles (LMV) and basolateral membrane vesicles (BLMV) were isolated from the equine colon using cation precipitation and differential centrifugation techniques. Characterization and LMV and BLMV origin and purity were performed using immuno-blotting analysis and enzyme assays. The uptakes of [14C]-butyrate were measured by rapid stop filtration technique.

## Results

Our data indicate that butyrate transport in both LMV and BLMV is stimulated by pH and bicarbonate gradients. Butyrate transport in LMV has Km constant and Vmax of 5.61 ± 0.45 mM and 614.32 ± 55 pmol/s/mg proteins respectively. Butyrate uptake was significantly impaired by phloretin and 4-CHC, the classical inhibitors of monocarboxylate transporter, and not by SITS and DIDS. At the Basolateral side, butyrate transport has Km constant of 12.2 ± 2.1.

mM and a Vmax of 1022 ± 84 pmol/s/mg protein. BLMV was markedly inhibited by SITS and DIDS however, phloretin and 4-CHC failed to influence butyrate uptake in the BLMV.

## Conclusions

These results indicate that butyrate transport is mediated via two distinguished transporters at the luminal and basolateral poles. In the LMV, butyrate transport is mediated via carrier protein transporter which may belong to the monocarboxylate transporter protein family.
